# Is there a relationship between static and dynamic ankle joint dorsiflexion during gait in people with a history of neuropathic ulceration?

**DOI:** 10.1186/1757-1146-4-S1-P47

**Published:** 2011-05-20

**Authors:** Anita Raspovic, Rogers Douglas

**Affiliations:** 1 Department of Podiatry and Musculoskeletal Research Centre, La Trobe University, Melbourne, Victoria, 3086, Australia; 2 School of Human Bioscience, La Trobe University, Melbourne, Victoria, 3086, Australia

## Background

The inclusion of limited joint mobility (LJM) as a risk factor for plantar neuropathic foot ulceration in diabetes is interspersed throughout the literature. This is commonly believed to occur through connective tissue thickening and stiffening, thereby reducing available static and dynamic motion below that required for normal foot function. High underfoot pressures are postulated to result, leading to increased ulcer risk. This project investigated this theory as it relates to ankle joint dorsiflexion in people with a range of lower limb complications due to diabetes.

## Methods

Fifty-six participants completed the study. Forty-one participants had diabetes and fifteen participants made up an age and gender-matched reference group (NOND). Of the diabetes group, ten had a history of past neuropathic ulceration (DNU), eighteen presented with peripheral neuropathy and no foot ulcer history (DWC) and thirteen had no lower limb complications (DNC). Maximum static ankle joint dorsiflexion was measured using the Lunge Test. Ankle joint kinematic data and plantar pressures were evaluated using the VICON^®^ motion analysis and PedarX^®^ mobile in-shoe systems respectively.

## Results

A trend of reduced static foot dorsiflexion existed in those groups with peripheral neuropathy (DNU / DWC) by an average of 3° to 7°. The 95% confidence interval of the mean difference between the DNU and DNC groups, for these measures, did not reach statistical significance however came close (6.89° mean diff, 95% CI: -17.13 to 3.36 left side; 5.44°mean diff, 95% CI: -13.13 to 2.24 right side). The 95% confidence interval of the mean difference for dynamic ankle dorsiflexion was also not statistically significant for the DNU and DNC groups (3.76° mean diff, 95% CI: -1.38 to 8.89 left side; 2.31°mean diff, 95% CI: -1.68 to 6.29 right side). Conversely to the static measures however was the trend for mean dynamic foot dorsiflexion used in gait to be approximately 3° to 4° greater in the DNU group compared to the DNC group. Importantly, the available static range of ankle dorsiflexion was not being fully utilised during gait. Ample range of additional dorsiflexion was available should it be required, to the order of 15° in the DNC group and 17° in the DWC group, on average. No correlation between measures of static and dynamic ankle dorsiflexion were found. In addition, no consistent relationship was detected between dynamic ankle dorsiflexion during gait and peak plantar pressure.

## Conclusions

The findings of this study question the validity of past theories whereby LJM is thought to be problematic through blocking dynamic motion requirements.

